# The good, the bad, and the blameless in parenting: a thematic analysis of discussions of childhood obesity on an internet forum

**DOI:** 10.1186/s12889-023-15314-6

**Published:** 2023-03-08

**Authors:** Terhi Koivumäki, Piia Jallinoja

**Affiliations:** Present Address: Tampere University, Tampere, Finland

**Keywords:** Childhood obesity, Parents, Parenting, Social media, Obesity stigma, Thematic analysis

## Abstract

**Background:**

Childhood obesity is affecting an increasing percentage of families globally. For families, obesity is often a tense issue, not least because of the negative stigma and cultural perceptions associated with it. Discussions around childhood obesity do not take place only at home or in healthcare, but increasingly on social media, such as Internet discussion forums. Our aim was to analyse how childhood obesity is discussed on a Finnish online discussion forum by parents of children with obesity and other commenters.

**Method:**

We gathered and analysed 16 discussion threads on childhood obesity taken from a Finnish Internet discussion forum, vauva.fi, between 2015 and 2021 (a total of 331 posts). For the analysis, we chose threads where the parents of a child with obesity took part. The parents’ and other commenters’ discussions were analysed and interpreted with inductive thematic analysis.

**Results:**

In the online discussions, childhood obesity was discussed mostly in the context of parenting, parental responsibility and lifestyle choices within the family. We identified three themes that were used to define parenting. In the theme of proving good parenting, parents and commenters listed healthy elements in their family’s lifestyle to show their responsibility and parenting skills. In the theme of blaming bad parents, other commenters pointed out mistakes in the parents’ behaviour or offered them advice. Moreover, many acknowledged that some factors causing childhood obesity were outside the parents’ influence, forming the theme of lifting the blame from parents. In addition, many parents brought up that they were genuinely ignorant of the reasons for their child’s overweight.

**Conclusions:**

These results are in line with previous studies suggesting that in Western cultures obesity – including childhood obesity – is typically seen as the individual’s fault and is associated with negative stigma. Consequently, counselling parents in healthcare should be expanded from supporting a healthy lifestyle to strengthening parents’ identity as being good enough parents who are already making many health enhancing efforts. Situating the family in a wider context of the obesogenic environment could ease the parents’ feelings that they have failed at parenting.

**Supplementary Information:**

The online version contains supplementary material available at 10.1186/s12889-023-15314-6.

## Background

Childhood obesity is a major concern in Finland and worldwide. In 2019, globally 38 million children under the age of 5 were overweight or obese, and the prevalence of childhood overweight among children and adolescents aged 5–19 has increased from 4% (1975) to 18% (2016) [1]. In 2020, in Finland, 29% of boys and 18% of girls aged 2–16 years were overweight or obese [2]. There is a large and fairly consistent body of evidence demonstrating that obesity in childhood has adverse consequences on premature mortality and physical morbidity in adulthood [3].

A central cause for the increase is the changes in eating and physical activity behaviours that have changed the energy balance [4]. Our environment promotes excessive food intake and does not encourage physical activity [5]. Due to several simultaneous social, technological and environmental changes [6], it has become harder for parents to ensure a healthy environment for their children. Given these developments and the multifactorial nature of childhood obesity, lifestyle change is not a straightforward issue. Previous studies among parents of children with obesity^1^ have shown that often the reasons for not following a healthier lifestyle are practical, such as a lack of time, resources, or uncertainty about how to maintain or achieve better lifestyle choices in the family’s everyday life and surroundings [7, 8, 9, 10]. Moreover, parents do not always recognize that their child is obese [11, 12]. Some parents feel that they already know enough about healthy lifestyles [7], and they feel that health and happiness are more important than weight [13]. Alternatively, they might acknowledge that their child is overweight, but do not perceive it as a health risk [14].

Some parents fear that treating their child’s obesity may harm the child [7, 15, 16] or that they will be blamed for the situation [16, 17]. Indeed, obesity is typically attached to many negative attributes and cultural conceptions, that have an impact on parents. More specifically, several researchers have highlighted the negative social attitudes related to overweight and obesity [18, 19, 20]. Consequently, a balance needs to be found between managing children’s obesity and considering the related psychosocial consequences.

In addition, parents of children with obesity may face stigmatization. When seeking help for their child, parents are faced with a double moral burden: they feel they are blamed and shamed for the child’s weight, but at the same time they fear the negative psychological effects of targeting the child’s weight [21]. Parents easily feel isolated and blamed for causing their children’s obesity and appreciate a supportive forum where they can share experiences [22]. Fear of stigmatization or being seen as bad parents can affect the readiness of parents to seek help or discuss the weight of their child in healthcare [21]. For the parents feeling stigmatized, anonymous discussions on online platforms, may be an alternative to discussions in healthcare encounters [23]. This study aims to explore one such platform and how childhood obesity is discussed there by parents and other commentators.

### Childhood obesity in social media

In contemporary societies, the Internet is a central platform where citizens search for information, inspiration, and advice on overweight, obesity, and weight management. More broadly, the Internet has increased citizen engagement with health-related issues, and various platforms have enabled citizens to search for information and support for their health-related concerns. Easy access to the Internet and its various discussion forums in Western countries, such as Finland, have made these platforms also part of many parents’ everyday lives [24].

There is little previous research on parents’ activity and argumentation on Internet forums in the context of childhood obesity. In a study based on two Australian online parenting discussion forums, four major motives for using online forums were found: seeking advice, sharing advice, social support, and making judgements [25]. On these Australian forums, parents perceived childhood obesity as a public health concern; however, many brought up having difficulties in implementing the lifestyle change messages in everyday life [9]. In 2004, in an anonymous Finnish Internet discussion, parents were mainly viewed as the primary cause of childhood obesity. Parents were negatively described as having “lousy” characters and being unable to create an “adequate” emotional bond with their children [26].

Many parents seek help from social media forums, and consequently, it is vital to understand parents’ online activity and arguments. Moreover, it is also essential to explore what kind of comments and answers these parents get and the tone of the overall discussion, because of the potential impact on others visiting the platforms. The present study fills this gap and aims to increase understanding of how childhood obesity is displayed in social media discussions. This study aims firstly to explore the discussion around childhood obesity on a Finnish discussion forum between parents having a child with obesity and people reacting to the parents, and to search for and analyse the repeated themes therein. Additionally, in the discussion we consider what additional knowledge our results provide about childhood obesity that can be useful in parents’ counselling in healthcare.

## Material and methods

### Vauva.fi discussion forum

The dataset was acquired from Mohawk, a commercial provider that comprehensively collects and records Finnish social media content. We had access to the discussion threads that had been active for 7 years prior to February 2021. Screening the online discussion forums on the database with keywords “child obesity”, “child overweight”, and “child weight” showed that on the vauva.fi platform, childhood obesity was discussed more frequently than on other platforms.

In Finland, the vauva.fi (*vauva* meaning “baby” in English) discussion forum has been one of the most popular forums for years. There are about 7 million visits every month, and it reaches over 1.5 million of Finland’s 5.5 million population [27]. The sub-forums on this discussion forum are mostly related to children and family life. Moreover, there is an “open topic” sub-forum, known as a support forum for mothers and women. On vauva.fi, anyone can read the discussions without registration. However, one must register in order to participate in the discussions, with the exception of the “open topic” sub-forum. There, the platform automatically generates the cybername “visitor” for each non-registered commenter. As there is no premoderation, the messages are published as they are. However, the administrator of Sanoma Media Finland Oy deletes all the messages that break the law, and in some cases, messages that are against good manners are moderated or deleted.

### Data collection

**Fig. 1:**
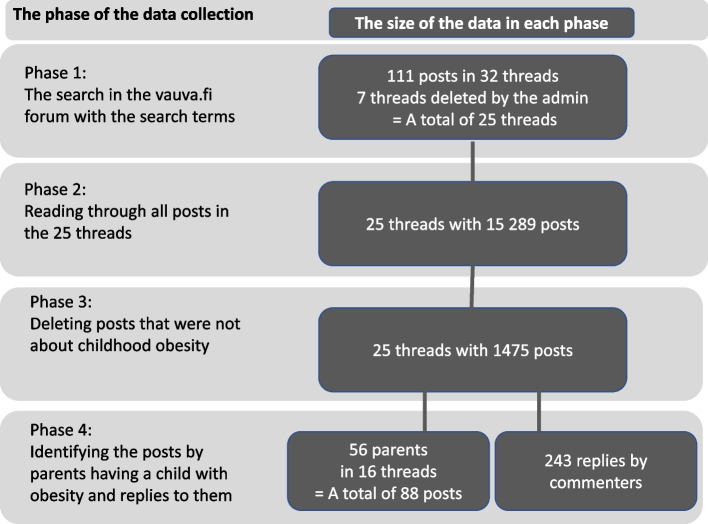
Steps in data collection

The purpose of the data collection was to locate relevant threads discussing childhood obesity, as well as discussions between parents having a child with obesity and other commenters in the forum. The data sampling included several steps (Fig. 1).

We used the search terms “child obesity” ~ 2, “child overweight” ~ 2, and “child weight” ~ 2 (= words were not more than two words apart from each other). With these search words, in the first phase 111 posts were found in 32 discussion threads. Seven of these threads were deleted by the administrator, leaving us with 25 threads. However, the threads contained more posts than these 111 posts. We estimated that to get a comprehensive picture of the discussions, it was essential to retrieve and read (by the first author) all the 15,289 posts in these threads. While reading all these posts, the inclusion criteria for the final data became as follows: the post contained a reference to childhood obesity, and it was either written by a parent of a child with obesity or it was a response to a post by a forementioned parents.

Using these criteria, we proceeded with the data extraction. First, we eliminated all the posts not covering childhood obesity, such as posts about obesity in general, or posts commenting only on specific foods – ending up with 1475 posts. Finally, to gain an especially parental perspective, we identified posts by parents having a child with obesity and posts reacting to the parents. There were 16 threads including parents’ posts, leaving us with a total of 16 threads and 331 posts. All these threads were on the “open topic” sub-forum.

To be categorized as a parent required that the person clearly expressed having a child with overweight or obesity. Few of the posts were identified as being posted by a mother and none by a father. As the gender of the parent was not evident in most posts, we named this group “parents”. People that were not identified as having a child with obesity, but commented on the parents’ posts, we defined as “commenters”. In the following analysis, the parents are marked as numbered parents. It was not possible to mark the commenters with separate numbers, because there they were mostly written under the automatic cybername “visitor”, instead of identifiable pseudonyms.

In addition, we use the term “obesity” – meaning both obesity and overweight – as we did not have exact information on the weight of the children. However, if the commenter used either word specifically, we used that term in the extracts. Likewise in the extracts, if the gender of the child or the parent is known, we refer to the person in a gender-specific way. Otherwise, we refer to them as s/he, the child, or the parent.

The length of the posts varied from a few words to over 400 words. The number of posts to parents also varied; some parents did not receive any answers to their posts, whereas some parents received nearly 50 responses. Moreover, the number of parents and other commenters taking part in the threads varied (see Attachment 1).

### Research ethics

As regards research ethics and related legislation in Finland, the present study does not fall under the scope of the Finnish Medical Research Act and Decree (488/1999) [28] and its obligation to be reviewed and approved by an ethics board, as our research does not include medical research. At Tampere University, where the research was conducted, there are no additional binding regulations to submit to a review process by an ethics board. Due to the lack of national- or university-level regulations on social media data in Finland, ethical review was not compulsory.

Due to this situation, we carefully applied impact-driven ethics, suitable especially for research with digital media [29]. This implies taking an active role in exploring the possible negative impact of the work for those whose posts we analyse. Moreover, we have considered the Finnish Data Protection Act 1050/2018 [30] and the Copyright Act (404/1961) [31], and concluded that the posts analysed here are anonymous and general enough in their style and content for it to be impossible to connect the individual posts to any single individual. Additionally, we followed the principles of *Ethical Decision-Making and Internet* [32] and the *Ethical principles of research with human participants and ethical review in the human sciences in Finland* [33].

### Data analysis

The posts were analysed with an inductive, thematic analysis. Our aim was to analyse the posts as social constructions of childhood obesity on social media platform. Thematic analysis is one potential method among qualitative methods enabling the analysis of interpretations and meanings behind the “surface” of the posts [34].

The thematic analysis was conducted following the phases presented by Braun and Clarke [35]: familiarizing ourselves with the data, generating the initial codes, searching for themes, reviewing the themes, defining and naming the themes, and finally producing the analysis. Regardless, the data process was not linear; rather the process required moving back and forth between phases and a constant return to the data.

**Fig. 2:**
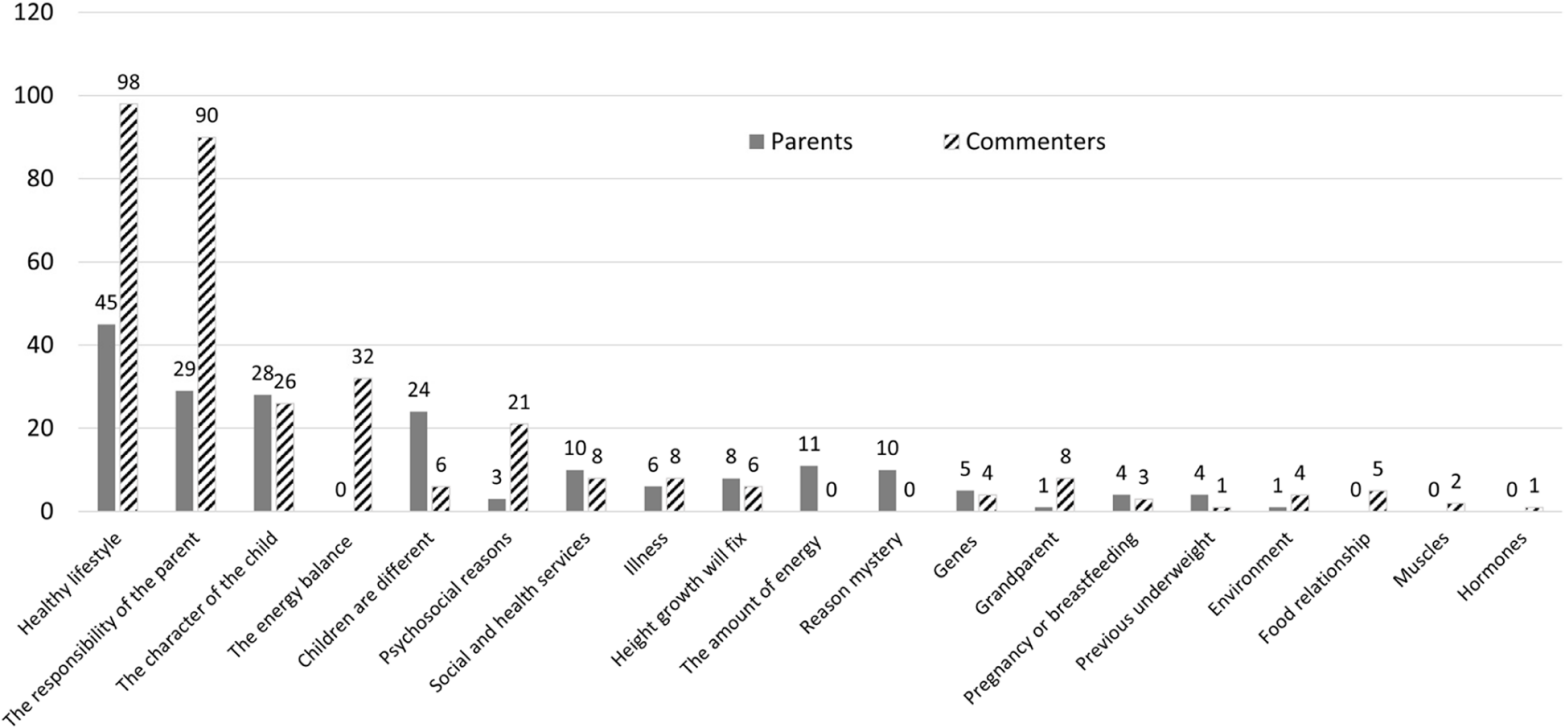
The initial codes and the frequency of their occurrence in the parents’ and commenters’ posts

When familiarizing ourselves with the data, we wrote down our initial thoughts and observations, and the first author identified that a major proportion of the posts concerned the reasons for and solutions to childhood obesity. Both authors discussed and verified this observation with several data examples. Next, sections of the posts where reasons and solutions were discussed were pinpointed, leading to a total of 512 sections of posts that were read and re-read several times by both authors. In this “repeated reading process” phase [35], 512 sections were further categorized into 19 initial codes related to reasons for and solutions to childhood obesity (Fig. 2). Some posts included reasons or solutions that fit several codes, and these were categorized into all suitable codes. For example, the following section was categorized with the codes “the character of the child”, “the responsibility of the parent”, and “healthy lifestyle”: *“In the afternoons, I can’t watch what they eat, but based on the remains, one can eat for example three bananas or five muesli bars at once (you can guess who)”* (Parent 41). The initial codes and how they were located in the parents’ and commenters’ posts are presented in Fig. 2.

The posts with their codes were collated into a single table to find connections between the different codes. There, with the posts under the same code grouped together, it was easier to notice repeated themes in the data. At first, it became clear that there was an emphasis on lifestyle choices, especially healthy or unhealthy eating. However, after returning to the codes and the data several times, we observed a repeated pattern of parenthood and estimations of its quality across the codes and the posts. This finding led us to create the main themes and the sub-themes. As mentioned, this phase required us to return to the data several times, and the first author pinpointed sections to keep the themes reliable and guarantee that they reflect what was expressed in the data. Braun and Clarke call this phase the refinement of the themes and it is part of the “reviewing the themes” phase [35]. Finally, during the fifth phase of the analysis, the names of the themes were discussed several times by both authors and then determined. Additionally, appropriate examples from the data were chosen to represent the themes.

Both authors familiarized themselves with the data in all phases. The first author made suggestions in every phase and these suggestions were discussed and modified by both authors several times. If there was any disagreement about the codes or themes, they were discussed, and the disagreement solved to reach a consensus.

The data were coded using AtlasTi 9.0, a qualitative data management software programme.

## Results

The commencement of the discussion threads about childhood obesity was often based on some negative news or article from the media, such as “Alarming research: Even children can have a fatty liver and high cholesterol – parents do not recognize a child’s overweight”, or written in an accusative tone: “Are parents really so blind that they cannot see the overweight of their own child?” Only two of the 16 sampled threads concerning childhood obesity were started by parents having a child with obesity. In addition, the number of parents in the threads varied. Eight threads included only one post from a parent. In other threads, more parents took part. For example, in several threads one parent first confessed that s/he has a child with obesity, followed by other parents sharing similar situations (Attachment 1).

What followed in the discussions revolved mainly around the reasons for childhood obesity and the related solutions. The reasons and solutions were mostly intertwined in the argumentation, so the presentation of a reason typically also implied a solution and vice versa. As already shown in Fig. 2, we found that childhood obesity and its causes and solutions were discussed chiefly in terms of a healthy lifestyle and parental responsibility and choices. Furthermore, in addition to the posts that directly focused on parents’ responsibilities, the question of parenting also lay at the core of most of the other posts, where parental responsibility was considered in relation to different family situations.

As regards parenting, we found three main themes – proving good parenting, blaming bad parents, and lifting the blame from parents – and their sub-themes (Table 1), which will be analysed in the following. In addition, we pay attention to the style of argumentation. The tone of the posts from commenters varied from supportive and counselling to accusing and blaming, as the titles of the themes and the following analysis and extracts will show.

**Table 1:** Main themes and sub-themes in childhood obesity related posts in the vauva.fi discussion forum

Main theme	Sub-theme
Proving good parenting	Making healthy choices Making an effort
Blaming bad parents	Making unhealthy choices Problems in the family environment
Lifting the blame from parents	Genes, biological factors, and illnesses Circumstances around the family Child’s behaviour and personality Reason unknown

### Proving good parenting

Good parents were presented as active, making good lifestyle choices, and trying to change the situation that had led to their child’s obesity. We found two ways of proving good parenting: by bringing up the healthy choices one has made and showing that one is at least making an effort.

#### Making healthy choices

It was very common to post about various things that had been done “right” in everyday life. These posts were mostly by parents, but also by some other commenters. In the discussions, a good parent was seen as one who is active in making healthy choices and avoids unhealthy options, especially unhealthy food choices, in the child’s everyday life. Parents attempted to convince others that even though their child was obese, they had done things that a good parent should do, such as providing their child with healthy rather than energy-dense foods and supporting a physically active lifestyle. Parents did recognize the importance of a healthy lifestyle, but often felt they had done all they could already.

*We do not go in for biscuits, cakes, and buns, nor do we drink juice every day. They do not drink sodas or [eat] chips,* etc. *they get those maybe 5 times a year. They exercise actively every day.* (Parent 14).

Another way to show good parenting was by using other, normal weight children in the family as proof of good parenting. These statements by parents were often accompanied by lists of lifestyle factors that promote health, as was done by the following parent.

*If just someone would find out the reason for gaining weight? We have four children, and only one of them is overweight. The whole family eats the same food and has the same habits with snacks. This child has no extra money to buy any additional food or snacks.* (Parent 15).

A few commenters listed reasons why there were no weight issues in their families. The commenters presented themselves as successful, good parents, setting an example to other parents of how things should be done.

*I have an 8-year-old and two other children. No-one lies the whole day with a mobile, they go out every day, because I make them, and they do not buy candy from the grocery store without permission, because I don’t give them money for that. And this is why their bellies do not grow.* (Commenter).

Comments on lifestyle were mostly in line with the official nutrition guidelines, but there were also some posts that included a different view about a healthy lifestyle, such as the strict restriction of bread or other carbohydrate sources.

#### Making an effort

Another way of presenting good parenting was showing one’s effort-making, trying one’s best to make good lifestyle choices, even when these efforts did not bring results. A good parent of a child with obesity was presented in the discussions as someone who is actively seeking a solution and is ready to ask for and receive advice. Parents brought up the efforts they had made and assured that they had spent time trying to reverse the child’s weight development.

*I can even make a vow that we have done more than many others for the welfare of our children.* (Parent 31).

Moreover, the commenters pushed and advised the parents to make an effort or ask for advice on how to manage the child’s weight. Parents did recognize the importance of a healthy lifestyle, but they often felt that they had already done all they could. Alternatively, some parents provided reasons why they could not implement sufficiently healthy choices in their own or their child’s everyday life. Parents wanted to present themselves as active parents who were trying their best. Simultaneously, the commenters expected the parents to try harder to change the lifestyles of the family.

*If a two-year-old eats an adult’s portion, how will s/he eat when s/he’s bigger. A horse’s ration? Your task is to ensure proper portion sizes, and the child may be thirsty and not hungry… It is the parents’ duty to see that the child eats enough, but not too much.* (Commenter).

### Blaming bad parents

The second repeated theme in the posts was the theme of blaming bad parents. Bad parenting was presented in two ways: first, by focusing on the unhealthy lifestyle of the family, and second, by highlighting the broader problems with the family and the parents’ inability to respond to the child’s emotional needs in the correct way.

#### Making unhealthy choices

Commenters were active in sharing knowledge and advice on how to eat healthily, avoid childhood obesity and raise healthy children. In these statements, there was a presumption that parents having a child with obesity were doing something wrong, especially in terms of offering unhealthy foods and not being assertive enough.

*You should change the hard fats to soft fats, add vegetables/berries/fruits, eat according to the plate model, emphasizing vegetables, eat less sugar and white wheat, change the pasta, bread, porridge to wholewheat products, eat less meat and prefer a more vegetable-rich diet. These things will get you forward. I suppose you cook food at home, so it should not be too much effort to make these changes. No one else is doing these changes, it is easy to make excuses.* (Commenter).

*This answer [referring to the post of one parent] sums up what is wrong in Finnish families. It is the lack of parental authority. An eight-year-old wants to lay down and stare at a mobile. And they LET the child do this, how can this be possible?* (Commenter).

In one discussion thread, a parent started defending her situation and commenters pointed out mistakes the parent had made. When this parent continued to defend herself and challenge other commenters, the commenters became more aggressive, suggesting that the parent had failed in parenting. In the end, the commenters no longer addressed their comments to the parent but started to discuss the situation of the family by themselves.

*Do others see how this one mother is defending her actions, even though the son is clearly morbidly obese? And how annoying is it, when such a reasonable discussion is filled up with this mother’s defensive talk? She really does not seem to believe that her child is in bad shape and is defending herself by lifting herself and her “fancy home cooking” onto a pedestal.* (Commenter).

*I think this one mother knows that she is at least partly to blame for her son’s considerable overweight. Otherwise, nobody would come here all the time to defend their situation so fiercely. Your son has to lose weight significantly to have a healthy life.* (Commenter).

It was very rare for parents to confess that they had done things wrong. In these few posts, parents expressed guilt and felt responsible for the child’s obesity.

*I feel guilty that I let my son gain weight*. (Parent 29).

*I don’t get why I really have not noticed this [child’s obesity] before. The school nurse said last autumn that we are going into the upper limits of the growth charts and the family should look at what it eats. The girl is 12 years old. She likes sugary stuff; I have tried to limit it. But she eats up to three plates of even basic food. How can I deny her when she is saying that otherwise she will stay hungry? Damn, I have been a bad mother.* (Parent 53).

#### Problems in the family environment

Another way of presenting bad parenting was by pointing out that the parents were creating a harmful family environment. Commenters suspected that the child’s obesity or eating was only a symptom of something else, such as depression, teasing, or simply boredom, and it was the parent’s responsibility to prevent the child from compensating for emotional needs with food. Again, the commenters blamed the parents for not meeting the emotional needs of their children and sometimes offered related advice, as in the following extracts.

*A healthy relationship to eating comes when eating in the child’s environment is healthy. If there are feeling-eaters around showing a model of vomiting-eating, that does not provide a healthy model for learning*. (Commenter).

*Have you offered your son anything else than food? Experiences, hobbies, being together, experiences in succeeding? Pleasures without food? Usually, if there is meaningfulness and passion in life, it can be anything from playing drums, dinosaurs, to camping, one does not need to binge oneself into a ball. Because then the food is in that situation where it belongs, and the meaning of life comes from somewhere else than the daily chocolate bar or ham sandwiches.* (Commenter).

In the extract below, a commenter is suggesting that some parents hide behind a rationalization that the child’s behaviour is unchangeable. Instead, s/he suggests that adults should look into the causes behind the behaviour and food preferences.

*[The explanation that] “the child is only greedy for delicacies” is often a simplified excuse for a situation where there are bigger problems in the background. Adults are easily blind to these, because they do not want to see the bad feelings of their own child.* (Commenter).

Most parents did not react to posts that accused them of being unable to fulfil the child’s emotional needs. Sometimes the parents did defend themselves, however, like the mother below.

*Honestly, I feel disgusting when you paint a picture in which my son is some kind of filthy and mentally bored outcast and us parents are people who have abandoned their child.* (Parent 31).

### Lifting the blame from parents

Finally, the reasons and solutions for childhood obesity were presented as being out of the parents’ control and hence the parents were blameless for their children’s obesity. We categorized these reasons and solutions into four groups: genes, biological factors, and illnesses; circumstances around the family; the child’s behaviour and personality; and reason unknown. Among the parents, the blameless parenting theme was intertwined with proving good parenting: often parents first presented their good parenting by listing things that they had already tried or done. Later, they stated that they would do more, but there was nothing left to do. The parents used the blameless parenting arguments more often, but commenters also gave parents absolution when they brought up causes of obesity that were not the parents’ fault and solutions that were out of the parents’ reach.

#### Genes, biological factors, and illnesses

Both parents and commenters acknowledged that there were various biological or medical factors behind childhood obesity, including hereditary, genetic and hormonal factors; a hefty body size; illnesses; and a slow metabolism, as the following extracts show.

*Children from the same family, eating the same food, can become different in body shape because of their genes.* (Commenter).

*There have been [health] inspections for years and s/he is medicated constantly. Outwardly, only weight is visible from the disease, although s/he has other problems caused by the disease, which are more serious than weight*. (Parent 32).

Even though some commenters identified these factors, such as diseases, as mostly outside the parents’ influence, they nevertheless often offered advice on a healthy lifestyle.

*If Prader-Will is excluded, then [the child’s appetite] could be a condition caused by fructose, in which satiety hormones and peptides do not affect the brain. Leave out the sugar and see if the situation levels out.* (Commenter).

In addition, the mother not eating sufficiently during pregnancy, breastfeeding too much and not breastfeeding at all were presented as reasons, too. These could also be interpreted to be under the parents’ influence and hence proof of bad parenting. However, here we categorized these accounts as examples of lifting the blame from parents, because especially the parents presented these issues as something that they could not have changed in their situation. For example, the mother below suggests the quality of her milk caused the child’s obesity.

*My child was born big and gained weight from long breastfeeding. My milk was like cream.* (Parent 49).

Some parents were in a situation where the child became obese after being underweight. These parents felt that the previous underweight was the reason behind the obesity, and often the concern about the child not having enough food led to a situation where the child got used to big portions and favourite meals.

*And then suddenly everything turned upside down. The girl started to eat everything, even too much. We did not prevent this, while we remembered this skeleton-thin girl, and if she now wanted 7 meat balls and two bread rolls, she was allowed to eat them. If she wanted a third bowl of cereal, she got it.* (Parent 6).

Moreover, some parents and commenters set their hopes on future height growth to solve the obesity problem. The commenters shared experiences when their own weight was normalized during height growth.

#### Circumstances around the family

There were relatively few posts where the social networks and social and health services were brought up as reasons for obesity. Both parents and commenters presented these as either a reason for childhood obesity or containing a potential solution to it. These environments were seen as ones over which parents had no influence.

Public institutions, such as child healthcare clinics and day care, were seen either as a reason or a solution to childhood obesity, both among parents and commenters. A good example of this is a lengthy thread where one parent suspected day care food to be the reason for the child’s obesity.

*Both my children are overweight, aged 4 and 5. They started gaining weight when they started at kindergarten and the consumption of bread and sugar bounced up significantly. In the kindergarten, they also swear by fat-free milk products.* (Parent 14).

The parent above received mostly disagreeing posts about blaming the day care, but also some posts agreeing that the day care was the reason for the obesity. In other threads, child welfare clinics and other experts were mentioned a few times as solutions for childhood obesity and mainly as places to seek help and advice. There were also posts expressing a wish to receive support from experts in healthcare. In the following extract, the commenter is optimistic about the help from the child welfare clinic, whereas the parent below is more sceptical.

*You should ask for help from the child welfare clinic to make food portion sizes correct.* (Commenter).

*In child welfare clinics, weight management issues are quite poorly taken care of. With my slightly chubby child, the advice was to add vegetables and use non-fat milk.* (Parent 8).

Grandparents were also mentioned as a reason for the child’s obesity in several posts, mostly in one thread. These were mainly responses to one parent who complained that their child was obese due to the grandparents’ treats. Some commenters questioned this explanation, whereas others supported it and shared similar experiences. In the next extract, one commenter shares similar experiences with her mother.

*My mom was the same with me and now she is also trying to fatten my own child. She just does not understand for some reason that you cannot take a bun or cake with coffee every day or maybe an adult can, but with a small child, it will definitely lead to a sugar spiral.* (Commenter).

A few comments were made also about the environmental influence on the child’s weight, such as chemicals and microplastics, Teflon pans, cosmetics, antibiotics, and intestinal flora. Interestingly, an obesogenic environment – such as the supply of energy-intense food and a car-dominated environment that does not encourage exercise – played no significant role in the posts. If a passive environment or energy-dense products were mentioned, it was presented in the context of the family’s everyday life as a challenge that should be solved by the parents or child.

#### Child’s behaviour and personality

In several posts parents or commenters presented a certain characteristic of the child as the reason for obesity, for example the child’s habit of overeating, preference for inactivity, or personality. Parents gave examples about their child’s character and commenters about families near them – both suggesting that some children had more challenging personalities causing obesity. It is noteworthy that not only the parents but also the commenters felt that sometimes the child’s qualities were beyond the reach of the parents’ efforts.

*While children are different. In my friend’s family, one child is chubby, two others are normal weight, even slim and sporty. There is a healthy lifestyle in this family, but this chubby child has a huge appetite for treats. I have noticed this when the child is visiting our home. At parties, this child hogs treats above everybody else, eats double the amount of food compared to others,* etc. *S/he is lazy and does not like to exercise. This family, I think, has a very normal attitude towards food, treats are not a forbidden fruit, but if they are offered without limits, this one child would eat them without end.* (Commenter).

Some parents identified that their child’s energy intake was too high, but they were unsure how to solve the problem, mainly because the child’s appetite was huge, and they did not want the child to suffer from hunger. For example, the following parent suspects that the child’s obesity is perhaps related to some innate characteristics that the parent cannot change:

*What can one do when the child is really reluctant to drink enough water, (s)he has a huge appetite, and is very greedy for sugary treats? Age 11, so s/he is eating also in other places besides the home*. (Parent 3).

The following parent brought up several unhealthy lifestyle components that they were unable to change because the child was reluctant to make them.

*How do I force that 11-year-old out for a run, when he does not want to? The rest of the family cycles to the playground or takes a walk in the forest, but this first-born stays at home on the couch with his cell phone. And every now and then goes to look for something to eat from the fridge. He is keen on snacks, but also loves bread, which s/he can eat a bag a day. Probably also buys snacks secretly from the shop.* (Parent 5).

#### Reason unknown

Commenters always had a reason or solution – or at least a suggestion – for the child’s obesity, but several parents expressed a genuine lack of understanding as to why their child was obese. If the cause of the obesity was considered unknown, there were no more solutions for the parents to search for. This ignorance was visible when the obesity of one child in the family was contrasted with the normal weight of the other children, as in the following extract:

*Not a single public health nurse nor doctor nor dietitian has come up with a reason why there are two slim children and one overweight in our family. We all eat the same food and there are sporty hobbies.* (Parent 36).

Another way of presenting ignorance was listing various typical reasons behind obesity and stating that none of them applied to their situation. Consequently, there were no solutions for their child’s obesity that could be adopted in the family, as the following parent presents:

*We eat normal home-cooked food (*e.g. *macaroni casserole and salad, water to drink with food), and when younger, he had a candy day. We do not purchase juice or soda! Still the boy has gone above the weight limits, big time. Tests have been taken, but no illness has been found that would explain this.* (Parent 44).

When parents explained that the reason for the child’s obesity was unknown, commenters again offered additional solutions and advice, mostly related to eating or exercise habits. Usually, parents did not reply to these posts, but if they did, and especially if they defended their choices, the commenters became more accusing in the tone of the theme of blaming bad parents.

## Discussion

This research aimed to explore the Finnish online discussion forum vauva.fi to identify, analyse and interpret discussions about childhood obesity. We have shown that childhood obesity and its causes and solutions were discussed mostly in the context of parental responsibility, the quality of parenting and a healthy lifestyle. We found three main themes that were used to define parenting: proving good parenting, blaming bad parents and lifting of the blame from parents.

The overall tendency in the posts was to associate good parenting with the family’s healthy lifestyle and consequent, lack of obesity among the children. As a result, parents having a child with obesity needed to prove their good parenting skills repeatedly on the discussion forum. A similar phenomenon was found in a study of parents of ill children: a good parent expresses both a personal sense of duty and devotion [36].

The commenters in our data provided a contrast to the parents’ posts, bringing up the theme of bad parenting – while only two of the parents stated that they were themselves bad parents. A previous study of social media discussions of childhood obesity by Kokkonen (2009) found, that parents were mainly viewed as the primary cause of the child’s overweight, and they were presented as having poor parental abilities [26]. Nnyanzi et al., have also reported that parents with normal-weight children view other parents – especially those whose children had a higher weight – as not doing things correctly [37]. Pulling together the themes of good and bad parenting, we conclude that our results reflect the cultural ethos where children with obesity are seen to signify parental failure and neglect, especially by mothers [38, 39]; the weight of one’s child has become a “litmus test of good mothering” [40].

Below, we reflect on our results with the concept of obesity stigma, meaning the tendency to attach negative attributes to obesity [41]. Several of the themes of our results reflect the cultural ethos where obese individuals are stigmatized. To begin with, the stigmatization of parents took place when parents of children with obesity were presented as failed parents who were unable to build a healthy environment for their children. Moreover, the theme of good parenting contained the potential for stigma, as good parenting was associated with the normal weight of the child – leaving other parents stigmatized as bad parents. Finally, our results suggest that children with obesity were stigmatized on the vauva.fi discussion forum too, as they were associated with negative stereotypes such as laziness and a lack of motivation and willpower.

On another level, the themes above reveal the strong tendency of modern societies to emphasize individual responsibility in health issues. Robert Crawford, for example, has pointed out that in recent decades in Western societies, there has been heightened concern about health issues from an individualistic perspective [42]. Individual-centred explanations for obesity have been reported among people taking part in lifestyle change interventions [43], among healthcare professionals [44] and among children [45]. Moreover, the centrality of lifestyle choices in our results is not a surprise, considering how obesity is presented as a result of the unhealthy lifestyles of individuals, especially a result of an unhealthy diet in the Western media, including the Finnish media [18, 19, 46]. There children with obesity are typically portrayed as lazy couch potatoes who should participate in the “war on obesity” along with their parents [19].

More detailed analysis of various media has shown the tones of media reporting on childhood obesity. On Twitter, childhood obesity was more often presented as a question of individual behaviour than of environment or policy [47]. Results from many countries reveal similar results: Boero (2009), has highlighted the tone of reporting on childhood obesity in the US media, which place the blame on mothers [40]. An analysis of American and Canadian parenting magazines showed that from the 1980s onwards parents have been advised to get their child eat more healthy food and less junk food, exercise more, and be less sedentary. Interestingly, since the early 2000s the advice has become more detailed, requiring a great deal more effort from the parents [48]. In the UK print media, childhood obesity has been related to individual-level drivers and solutions more frequently compared to societal-level drivers and there parents were mentioned frequently. [49]. Our analysis of online discussions of childhood obesity fit with this overview of how childhood obesity is presented in the Western media.

Blameless parenting was the third way of discussing childhood obesity, and it diverged from the strong tendency to define parenting by the lifestyle habits of the family. One type of blameless parenting was present in posts where parents expressed confusion about their child’s obesity despite the parents’ good intentions and healthy family habits. Various genetic and medical factors, the child’s behaviour and personality, and the practices of grandparents or day care centre in building eating patterns were brought up as causes of obesity. Others have also observed similar themes. Among the parents studied by Syrad et al., weight was occasionally attributed to “inherited factors”, such as genetics or puppy fat [13]. Another study found that mothers felt that one reason why they were not able to provide healthy food for the child was that the child had special needs or food preferences [50]. Lastly, among American parents, the child’s dietary or activity preferences were seen as an obstacle to the parents’ efforts to guarantee the child’s healthy diet [10].

It is noteworthy that what has been termed the obesogenic environment [5]- meaning modern technologized societies with the constant use of television and computers, a sedentary lifestyle, and easy access to energy-dense foods and large portion sizes - was not mentioned in the discussions. Although mobile phones were mentioned in the context of family life, there was no criticism of the social or cultural system as a cause for children’s greater weight. This was rather surprising given that this environment has been widely reported and discussed in the Western media since the early 2000s. Instead, there was a strong tendency to blame the parents for the child’s obesity.

Another obesity- related new approach we did not find in the threads, is the acceptance of bodies of all sizes, such as “body positivity” or “Health at Every Size”. These perspectives on obesity have been increasingly visible in the media in Finland [51] and elsewhere, although less so regarding children. It remains to be seen if these discourses of obesity that expand beyond individual responsibility, will change the online discussions on childhood obesity from blaming and stigmatizing towards a broader understanding of childhood obesity.

## Strengths and limitations

Anonymous social media data have their strengths and limitations. There is no way of verifying or clarifying the stories in the posts, and some of the comments might be written sarcastically. In addition, the absence of facial expressions and body gestures may make it more difficult to interpret the posts correctly, especially if there was sarcasm. However, regarding these concerns, our results are in line with previous research on perceptions of childhood obesity among parents, utilizing online discussion forum data [9, 25, 26] and with qualitative research conducted with interviews among parents [13, 37, 50] and focus groups [52].

Social media as a source of research data offers new opportunities for researchers to explore and observe citizens’ opinions, activities and interactions on different topics [53]. On anonymous Internet forums, some participants may communicate their difficulties in more intimate ways, compared to face-to-face interviews or observation encounters in healthcare. Seale et al. [54] have shown that interviewees typically produce retrospective accounts related to the topics of preplanned questions, whereas on the Internet, the emphasis is on participants’ more current experiences. Therefore, social media as a source of research data have the potential to provide additional knowledge on various phenomena.

The present research adds to the understanding of perceptions of childhood obesity by analysing the discussion and interaction between parents and other commentators in an online discussion forum. Many parents read online discussions and hence are affected by how obesity and parenthood are discussed there. Additionally, Internet forums are not separate from the rest of life, and consequently, they reflect the broad attitudes towards obesity in our cultures. By knowing the cultural conceptions parents confront in online forums, healthcare professionals may gain insights into how to understand parents’ concerns and reactions better.

The strength of this research is that we were able to examine parents and other commenters separately, as well as the communications between them. In particular, the online argumentation of parents of a child with obesity has been under-studied thus far, so our study produced new information on parents’ perceptions of childhood obesity. In addition, we were able to analyse more broadly the attitudes towards families that include children with obesity.

## Conclusions for healthcare

The result of our study may also be used in the development of healthcare. First, we showed that parents who take part in online discussions of childhood obesity are frequently judged as being poor parents or even as having failed in parenting. These negative experiences can weaken the willingness to seek help from support services [55] and may cause heightened sensitivity to well-intentioned questions and comments about the child’s weight from healthcare personnel. Consequently, healthcare professionals need to be careful not to reinforce the cultural model that stigmatizes children with obesity and their parents. One possibility to do this involves inclusive, affirmative, and health-oriented messages and practices in healthcare encounters that are not weight-focused [56].

Second, our results showed that many parents struggle to find ways to reverse their child’s weight development, and parents often found these struggles frustrating. However, instead of being “educated” about healthy lifestyles, parents could benefit from tools that reduce frustration when trying to change children’s food, screen, or sleep habits [52] and promote positive relationships between parents and children [57]. We also found that parents were eager to report how they prepare healthy foods for their children and push them towards physical activity. These activities should be noted in healthcare, to strengthen the parents’ sense of accomplishment. Overall, counselling could be developed in a more family-focused direction.

Finally, we showed that the obesogenic environment was not presented as an underlying factor of childhood obesity in the discussions. Discussing the pressures of the environment in healthcare could help some parents shed some of the burden and guilt they are currently feeling.

Finally, the healthcare worker’s ability to face and accept the parents’ emotions and offer support for parental skills could help parents to cope in their present situation with the child’s weight.

## Supplementary Information

**Additional file 1. **Attachment 1. The list of discussion threads included in the data and number of parents (*n* = 56), their posts (*n* = 88), and commenters’ posts (*n* = 243) in the threads.

## Data Availability

All posts used for the analysis are available from the corresponding author upon request. The database Mohawk is a commercial provider that comprehensively collects and records Finnish social media content and is only available to customers. The vauva.fi (https://www.vauva.fi/) discussion forum is publicly accessible; it is not password protected and the posts may be read by anyone.

## Footnotes

1 In this article, the term “obesity” incorporates the term “overweight”, and the authors use person-first language when describing children with overweight or obesity.
