# Supplementary material for: The good, the bad, and the blameless in parenting: a thematic analysis of discussions of childhood obesity on an internet forum
Source: BMC Public Health. 2023 Mar 8;23:452. doi: 10.1186/s12889-023-15314-6 (PMC9993749; doi:10.1186/s12889-023-15314-6)
Supplement: Supplementary file 1 — Additional file 1. Attachment 1. The list of discussion threads included in the data and number of parents (n = 56), their posts (n = 88), and commenters’ posts (n = 243) in the threads. [file 12889_2023_15314_MOESM1_ESM.docx]

**Attachment 1.** The list of discussion threads included in the data and number of parents (n=56), their posts (n=88), and commenters’ posts (n=243) in the threads.

| The heading of the discussion and date of the first post | Parent ID/the total amount of parents’ posts in the thread | The total number of commenters’ posts in the threads |
| --- | --- | --- |
| 1. Why is somebody else’s obesity concerning to you? / 30 September 2020 | ID 1 / 1 | 2 |
| 1. Estonian people are shocked about obesity in Finland / 22 July 2019 | ID 2 / 1 | 1 |
| 1. Alarming research: Even children can have a fatty liver and high cholesterol – parents do not recognise a child’s overweight / 18 May 2019 | ID 3–8 / 10 | 17 |
| 1. Research: Over half of parents do not recognise their child’s overweight / 10 May 2019 | ID 9–12 / 4 | 4 |
| 1. Granny is trying to fatten the child who is already on the edge of overweight?! (Parent) / 8 November 2018 | ID 13 / 1 | 41 |
| 1. YLE: Overweight of children exploded! Should the parents be blamed for causing trauma? / 21 September 2018 | ID 14–22 / 12 | 26 |
| 1. XL generation – childhood overweight / 27 April 2018 | ID 23 / 1 | 1 |
| 1. After being in a cottage with an overweight family, I understand why my family is not overweight / 14 July 2018 | ID 24–25 / 3 | 6 |
| 1. Childhood obesity in small children has become common, why it is not intervened in more strictly? / 14 July 2018 | ID 26–29/ 8 | 31 |
| 1. Are parents really so blind that they cannot see the overweight of their own child? / 18 September 2017 | ID 30–42 / 27 | 55 |
| 1. Childhood overweight / 26 July 2017 | ID 43–46 / 4 | 2 |
| 1. Childhood obesity and parents’ responsibility / 7 August 2008 | ID 47–52 / 6 | 15 |
| 1. Childhood obesity is a ticking timebomb – a fifth of school children are overweight / 19 October 2016 | ID 53 / 1 | 0 |
| 1. The obesity of your own child – how can I help? (Parent) / 24 May 2017 | ID 54 / 6 | 41 |
| 1. Every third European child aged 6–9 is overweight / 31 May 2016 | ID 55 / 2 | 1 |
| 1. Do you think that childhood overweight is cute? / 26 January 2016 | ID 56 / 1 | 0 |
